# Dendrimer: An update on recent developments and future opportunities for the brain tumors diagnosis and treatment

**DOI:** 10.3389/fphar.2023.1159131

**Published:** 2023-03-16

**Authors:** Monika Kaurav, Sakina Ruhi, Husni Ahmed Al-Goshae, Ashok Kumar Jeppu, Dhani Ramachandran, Ram Kumar Sahu, Ashish Kumar Sarkar, Jiyauddin Khan, Abu Md Ashif Ikbal

**Affiliations:** ^1^ Department of Pharmaceutics, KIET Group of Institutions (KIET School of Pharmacy), Delhi NCR, Ghaziabad, India; ^2^ Dr. A.P.J. Abdul Kalam Technical University, Lucknow, Uttar Pradesh, India; ^3^ Department of Biochemistry, IMS, Management and Science University, University Drive, Shah Alam, Selangor, Malaysia; ^4^ Department of Anantomy, IMS, Management and Science University, University Drive, Shah Alam, Selangor, Malaysia; ^5^ Department of Pathology, IMS, Management and Science University, University Drive, Shah Alam, Selangor, Malaysia; ^6^ Department of Pharmaceutical Sciences, Hemvati Nandan Bahuguna Garhwal University (A Central University), Chauras Campus, Tehri Garhwal, Uttarakhand, India; ^7^ School of Pharmacy, YBN University, Ranchi, Jharkhand, India; ^8^ School of Pharmacy, Management and Science University, Shah Alam, Selangor, Malaysia; ^9^ Department of Pharmaceutical Sciences, Assam University (A Central University), Silchar, Assam, India

**Keywords:** dendrimers, brain tumor, brain tumor imaging, brain targeting, theranostics

## Abstract

A brain tumor is an uncontrolled cell proliferation, a mass of tissue composed of cells that grow and divide abnormally and appear to be uncontrollable by the processes that normally control normal cells. Approximately 25,690 primary malignant brain tumors are discovered each year, 70% of which originate in glial cells. It has been observed that the blood-brain barrier (BBB) limits the distribution of drugs into the tumour environment, which complicates the oncological therapy of malignant brain tumours. Numerous studies have found that nanocarriers have demonstrated significant therapeutic efficacy in brain diseases. This review, based on a non-systematic search of the existing literature, provides an update on the existing knowledge of the types of dendrimers, synthesis methods, and mechanisms of action in relation to brain tumours. It also discusses the use of dendrimers in the diagnosis and treatment of brain tumours and the future possibilities of dendrimers. Dendrimers are of particular interest in the diagnosis and treatment of brain tumours because they can transport biochemical agents across the BBB to the tumour and into the brain after systemic administration. Dendrimers are being used to develop novel therapeutics such as prolonged release of drugs, immunotherapy, and antineoplastic effects. The use of PAMAM, PPI, PLL and surface engineered dendrimers has proven revolutionary in the effective diagnosis and treatment of brain tumours.

## 1 Introduction

Cancer is currently associated with extremely high mortality and morbidity rates worldwide. The World Health Organization (WHO) predicts that early deaths will increase by 70% in the next 20 years (Cancer, 2022). In 2015, cancer was the cause of death for 8.8 million inhabitants around the world, and this number is expected to increase to 12 million by 2030. Although cancer incidence is lower in low- and middle-income countries (LIMICs), overall cancer mortality is much higher in LMICs, especially among those under 65 years of age. For this reason, human development and wellbeing are affected by cancer (Kesharwani et al., 2015; Thakur et al., 2015; Dwivedi et al., 2016).

A high mortality rate is associated with this type of disease when the metastatic growth is uncontrolled in the central nervous systems (CNS) (brain, spinal cord) (Sacks and Rahman, 2020). Approximately 25,690 cases of primary malignant brain tumors are diagnosed annually, 70% of which originate primarily in glial cells (Ostrom et al., 2021). World health organization (WHO) has classified brain tumors into four grades, ranging from I to IV depending on severity. Glioblastoma (GB), referred as type IV glioma, which is severe and invasive carcinoma with a survival rate of 5.1% (Tadros and Ray-Chaudhury, 2020; Katano and Yamashita, 2022). The radiation therapy, surgery and systemically administered chemotherapy are the three main techniques used in the conventional treatment of cancer. According to them, a median survival of 9 months is possible, with a survival rate of about 10% of 2 years. However, systemic chemotherapy has limited efficacy in brain tumors due to minimal drug uptake into the tumor cells, drug metabolism within tumor cells and intrinsic sensitivity of tumor cells (Zaal and Berkers, 2018; Tan et al., 2020; Stine et al., 2022).

Patients with multiple drug-resistant gliomas are more likely to have poor prognosis as they cannot be completely removed surgically and there is a possibility for a new primary tumor to form after surgery. Major challenges include the complexity and heterogeneity of glioblastoma (GB) molecular biology. As a result, the prognosis could differ significantly for every patient receiving the same treatment. Adjuvant chemotherapy and radiotherapy (RT) are primarily utilized to treat GB whereby both therapies can cause genotoxicity (Thakur et al., 2015).

Blood Brain Barrier (BBB) poses a challenge to the efficient treatment of brain tumours by preventing the transfer of drugs to the affected area. The BBB is a very important part of how drug molecules move from the bloodstream to the brain. It also prevents toxins and large and higher concentrations of therapeutic drugs from entering the brain microenvironment (Thakur et al., 2015). The main physiological barriers at the brain surface include the choroid plexus, arachnoid plexus and blood vessels. Other challenges include non-specific binding, bioavailability of drugs and imaging agents (Kesharwani et al., 2015).

Numerous studies have found that nanocarriers are effective in treating brain diseases. Drug distribution in the brain can be divided into two categories: First, bypassing and evading the BBB due to the different architectures, and second, targeting the drug across BBB vai utilization of characteristics of polymeric nanocarriers. Nanocarriers offer special benefits for drug delivery. The optimal physicochemical characteristics, such as solubility, particle size, potential, and shape, help to increase pharmacokinetics and biodistribution. Moreover, surface modification may boost medication accumulation in the target tissue to enhance the therapeutic effect. Additionally, the nanocarriers have a particular drug release characteristic that enhances drug concentration in the target site and decreases drug concentration in the non-target site, minimising the likelihood of unfavourable reactions. Moreover, it is simple to mix treatments with nanocarriers to generate synergistic effects. Hence, nanocarriers offer a good platform for the investigation of medications that target brain tumours. Few most commonly utilized nanocarriers are nanoparticles, liposomes, quantom dots, magnetic nanoparticles, dendrimers, micelles, carbon nanotubes, nanoemulsions, solid lipid nanoparticles, etc., (Sahu, et al., 2021; Yeini et al., 2021; Bhatt et al., 2022; Grover et al., 2022; Setia et al., 2022).

Dendrimers consist of either chemical conjugation of terminal functional groups on the drug surface or physical encapsulation of drug molecules in the internal cavities of the dendrimer (Kesharwani et al., 2015). The efficient passage of therapeutic molecules through the BBB can be attributed to their lipophilicity. Based on past studies, dendrimers are excellent solubilizers, particularly for macromolecules. Positively charged dendrimers, which consist of enmeshed Nanoparticles (NPs) with mucus are known to have increased cellular uptake when they are associated with mucus. In a recent study, the drug bortezomib was found to be less toxic when loaded into dendrimers that are acid and pH resistant and have the ability to altered the drug release. Therefore, this treatment shows great promise for the treatment of malignancies (Singh et al., 2019). Modifying the external features of dendrimers can be considered as a cost-effective way to acquire new capabilities. Modifying the surface of dendrimers would alter their biopharmaceutical properties, such as improved biocompatibility, release kinetics, targeting to the BBB or brain tumor, and delivery of bioactive and imaging agents through the BBB (Zhu et al., 2019).

This review summarizes the current status of brain tumors, available treatments, major challenges in the application of diagnostic and therapeutic nanocarrier systems and aspirations for future research. The architecture, species, physicochemical and biological properties of dendrimers, and synthesis methods are described in great detail. In addition, the pharmaceutical delivery and brain tumor imaging using dendrimer-based nanovesicles are highlighted as well. The article aims to provide information on the potential and problems associated with effective brain tumor targeting for therapy and diagnosis in a clear manner that can be understood by all. Finally, the limitations that dendrimers face in clinical applications and ways to overcome them will be addressed. The focus of this review is on recent therapeutic developments using dendrimers for brain tumor targeting and imaging.

## 2 Dendrimers and their characteristics

The current circumstances and obstacles related to brain tumors make it difficult for researchers to develop an efficient technique to detect and treat brain tumors. For this reason, it is highly recommended to develop a safe and effective delivery system that protects the payload and increases the effectiveness of the drug. Furthermore, this developed system must have improved drug release and offers reduced toxicity in the non-targeted organs due to aggregation of the charged cytotoxic materials. Although various nano-delivery systems have been utilized in recent decades, dendrimers have emerged as the leading system in brain tumor treatment and imaging due to their immense potential alongside existing nanocarriers (Akhter et al., 2013). Dendrimers are polymeric structures range of 1 nm–10 nm size that are firmly established, spherical, macromolecular, hyperbranched, three-dimensional, and multivalent., which give them better flexibility and monodispersibility (Chenthamara et al., 2019). With a large number of hydrophobic pockets, dendrimers are able to encapsulate a variety of bioactive substances, allowing for regulated and sustained release of drugs (Noriega-Luna et al., 2014; Choudhary et al., 2017; Rahman et al., 2020).

## 3 Structure of dendrimers

**FIGURE 1:**
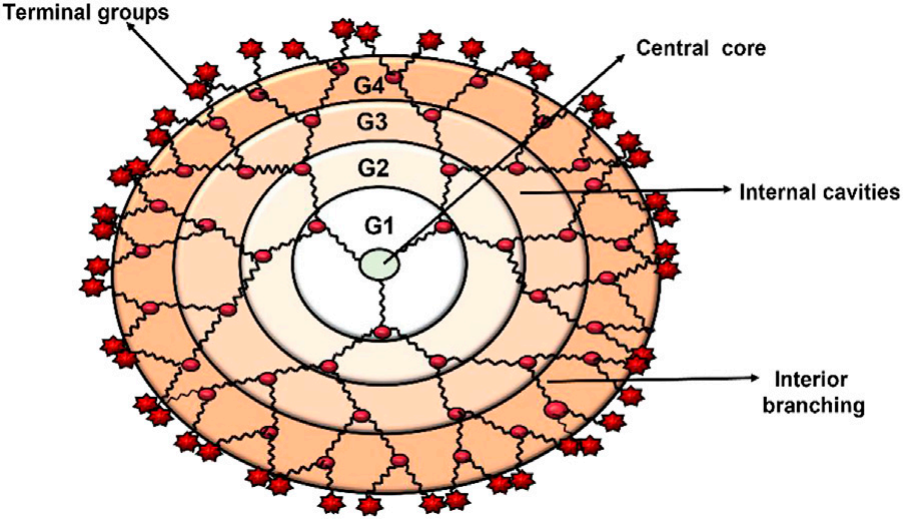
Dendrimer architecture at its most fundamental level.

**FIGURE 2:**
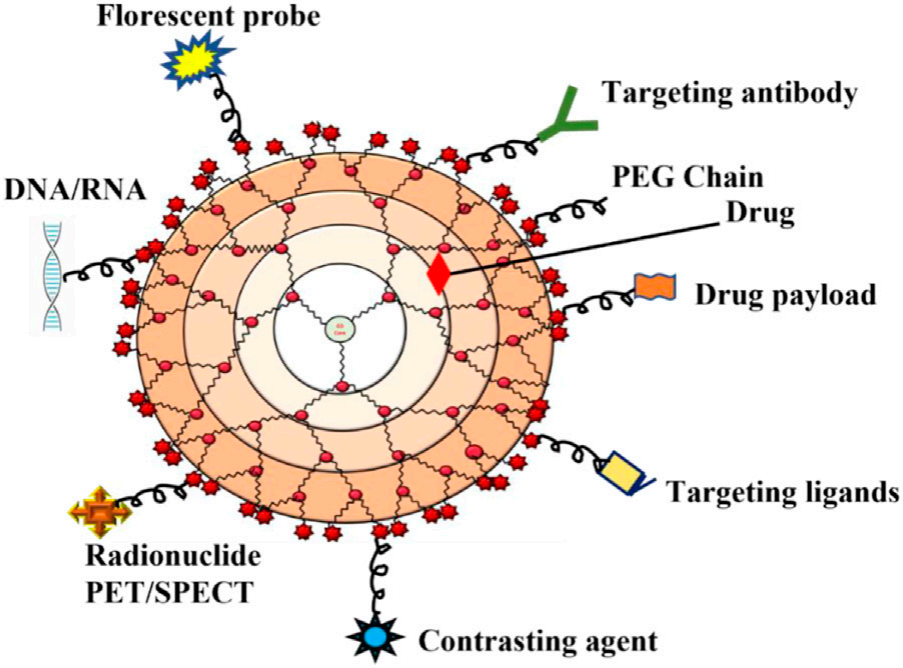
Schematic diagram that indicates the application of dendrimers in various biomedical fields.

Dendrimers are composed of a central core, branches (dendrons), and terminal functional groups (Figure 1). The attachment of the branches is made possible by the central nucleus, which consists of a single atom accompanied by at least one functional group. The branches (dendrons) arise from the atomic units of the core and bridge among themselves repeatedly at least one branching junction, resulting in an organized, radially concentric, layer-based geometric structure known as generations. Thus, the radially concentric layer is created by bridging between the branching units, which are referred as “generation (G)” (Birdhariya et al., 2015). As the generations are repeated, dendrimers of higher generations with spherical shape are formed, namely, the first generation (G1), the second generation (G2), the third generation (G3) and so on. The defined area formed between the dendrons is protected by a surface decorated with a multivalent end group. This empty area is used to encapsulate various bioactive materials and bio-imaging agents (Klimova et al., 2018; Tomalia et al., 2020). The loading efficiency of dendrimers would increase with every generation level. For example, cationic amino groups on the surface of higher generation cationic dendrimers would contribute to better DNA binding and cellular uptake by transforming the complex into nanoscale polyplexes (Noriega-Luna et al., 2014; Kesharwani et al., 2015; Tomalia et al., 2020). Therefore, the three main domains of the dendrimer architecture can be used for drug delivery, molecular sensors, genetic materials, enzymes, and bioimaging applications (Figure 2).

## 4 Methods of dendrimer synthesis

Dendrimers are compact spherical structures formed by high degree branching of polymers (Shi et al., 2006). In most cases, their synthesis involves the repetitive attachment of monomers to a central core that has multiple functional groups. Several different functional groups form the core of the structure. The addition of monomers to each functional group produces the next dendrimer and forms end groups for the next reactions (Walter and Malkoch, 2012; Mittal et al., 2021). The size of the dendrimers increases with packing after each generation and finally reaches its densely packed spherical structure with maximum size.

Depending on the goals of the study, either convergent or divergent strategies can be used to synthesis dendrimers (Šebestík et al., 2012; Kalhapure et al., 2015; Malkoch and García-Gallego, 2020). In the divergent method, the activation of the functional groups of the multivalent surface occurs in the first step and the addition of monomer units for continuous branch elongation begins (Jain et al., 2012; Kesharwani et al., 2015; Chauhan, 2018; Sherje et al., 2018; Janaszewska et al., 2019; Kumbhar et al., 2021; Sheikh and Kesharwani, 2021). The advantages of divergent methods are the possibility of surface modification and the ability to construct dendrimers with different physiochemical properties. Meanwhile, the convergent technique for the synthesis of dendrimers begins with the attachment of functionalized monomers to the interface where the dendrons are to be joined together to produce the finalized dendrimer architecture. In this process, the dendrons are linked together to form the dendrimer. Compared to the divergent method, which often results in incomplete branching, the main advantage of this method is the low error rate in the final structure (Cammidge, 2003; Svenson and Tomalia, 2005).

## 5 Dendrimer physical and biological properties

Dendrimers are spherical macromolecular structures at the nanoscale connected to various branched structures. They are best used for drug delivery and imaging. Conventional polymeric carriers are disperse, while dendrimers are mmonodisperse and have well-defined chemical structures. Furthermore, dendrimers’ unique framework enables them to be loaded with therapeutic agents *via* covalent conjugation or electrostatic adsorption (Du et al., 2013). Dendrimers normally resemble a set of biological structures in terms of size. For example, the fifth-generation polyamidoamine dendrimers (PAMAM) resemble hemoglobin in size and shape (5.5 nm) (Esfand and Tomalia, 2001).

The surface group of dendrimers can be either positive, negative, or neutral, which affects which group is best suited for drug transport. Compared to neutral or anionic-charged dendrimers, negatively charged dendrimers do not cause as much cell loss or hemolysis as positively charged dendrimers do (Malkoch and García-Gallego, 2020). In lieu of this, PEGylation would alter zeta-potential, plasma attainment and even distribution *in vivo* to overcome this issue. It is extremely difficult for free gene molecules to reach the targeted cells *in vivo* which is a major obstacle in gene therapy (Vannucci et al., 2013). Electrostatically, the positively charged dendrimers would be attracted to the negatively charged polynucleotides, leading to the formation of stable dendriplexes. After being taken up by the cell, these dendriplexes are unloaded into endosomes due to the sponge effect, which stimulates gene transcription in the cell (Sonawane et al., 2003; Perumal et al., 2008). Dendrimers can protect nucleotide substances from degradation, allowing the penetration of nucleic acids into the cells and maintaining the biological activity of gene molecules when they are utilized as gene delivery carriers.

## 6 Types of dendrimers

Developing an optimal delivery method for the treatment of brain tumours is challenging for researchers and clinical investigators, as there are currently many obstacles in this area (Dande et al., 2006). These limitations further necessitate the use of a safer and more effective carrier that protects the payload from degradation, penetrates the targeted area and increases the efficacy of the drug molecules. In addition, the carrier must also be able to regulate or optimize the release of the drug. Dendrimers, which have emerged in recent decades with numerous nanocarriers, are being touted as the stars on the current horizon because they are multitasking and flexible (Chis et al., 2020). Dendrimers, which possess unique properties such as nanosize, defined composition, and programmable surface functions, have been extensively explored for brain tumor therapy. Table 1 explains the basic properties of the different types of dendrimers and their advantages and disadvantages.

**TABLE 1:** Basic characteristics of different types of dendrimers.

Type of dendrimer	Characteristics	Advantages	Limitations	Drug/gene used	References
PAMAM Dendrimers	Hyperbranched, unparalleled molecular uniformity, narrow molecular weight distribution, defined size and shape characteristics, multifunctional terminal surfaces	Biocompatible, water soluble, non-immunogenic, high loading efficiency, Improved biological stability, cell uptake and intracellular trafficking and pharmacokinetics, immune modulator, glucose scavenger, alter cell signalling pathways	Prolonged administration cause organ and tissue toxicities	Luciferase siRNA, angiopep-2 peptides, Epirubicin Let-7g miRNA, Fibrin-binding CREKA, (glioma homing peptides), Biotin Pyridoxal, p42-MAPK siRNA, KLAK, Bcl-2 and VEGF siRNA, anti-GFP siRNA, Apoptin, etc.	Xu et al. (2016), Zarebkohan et al. (2016), Xu et al. (2016), Shi et al. (2020), Igartua et al. (2018), Sharma et al., 2018, Bae et al., 2019, Ban et al. (2021), Fana et al. (2020), Bae et al. (2021), Sharma et al. (2021), Li et al. (2022)
PPI Dendrimers	Highly branched, well defined size, narrow dispersity, ease of terminal end group modifications	controlled release, improved tumor penetration and bioavailability, greater gene transfection efficiency, reduced adverse effects, Non-immunogenic, amorphous, no notable toxicity, due to adjustable pore size able to encapsulate and release kinetics	Low hydro solubility, toxicity, haemolytic effect	Paclitaxel, Docetaxel, methotrexate, pORE-TRAIL, SiRNA, etc	Gajbhiye and jain (2011), patel et al. (2012), Somani et al. (2014), Patel et al. (2016), Noske et al. (2020)
PLL Dendrimers	Well organized three dimensional globular chemical architecture, high monodispersity, precise size, polycationic dendrimer having number of surface amines thus able to bind with polyanions (nucleic acids) *via* electrostatic interaction	Gene carrier due to excellent condensation potential with oligonucleotides, good biocompatibility, water solubility, biodegradability, and flexibility, safer as compared to other dendrimers, inherent antibacterial, antimicrobial, antiviral, etc., properties	Low stability	Doxorubin, methotrexate, Gemcitabine, aptamers, Camptothecin, Docetaxel, Fluorouracil, DNA,SiRNA, Imaging agents, diabetic and cardiovascular drugs, etc	Janiszewska et al. (2016), Hegde et al. (2019), Zhu et al. (2019), Gorzkiewicz et al., 2020
Carbosilane Dendrimers	Defined structure with terminal cationic and anionic groups, non-functional siloxane external shells	Non-toxic, good biocompatibility, good thermal stability, increased half life, bioavailability	toxicity	anti-cancer therapy, immunotherapy, drug delivery, and gene therapy	Gomez et al., 2013, Rabiee et al. (2020), Rodríguez-Prieto et al. (2016), Strasak et al. (2017), Perise-Barrios et al. (2015)
Phosphorus Dendrimers	Contains phosphorous atom at each branching point along with hydrophobic surface and hydrophobic backbone	Diverse synthesis methods available, high ability to stabilize and to complex with preapoptic SiRNA and increase uptake upt 100%, high yield, water solubility, improved PK and PD of drugs and conjugates, less systemic toxicity	Toxicity issues	small molecules, peptides, siRNAs, mRNAs, anticancer and antitubercular drugs	Shcharbin et al. (2013), Chis et al. (2020), Migrani et al. (2021a), Migrani et al. (2021b), Migrani et al. (2022), Posadas et al. (2022)
Peptide Dendrimers	wedge-like branched macromolecules having peptidyl branching core and/or covalently attached surface functional units	biocompatibility, diversity and multifunctionality, self-assemble nanosized structure	low hydrosolubility and high non-specific toxicity	biomedical diagnostic reagents, protein mimetics, anticancer and antiviral agents, vaccines, drug and gene delivery vehicles	Stalmans et al. (2014), Lalatsa et al. (2014), Xie et al. (2022), Sowińska et al. (2022), Cieślak et al., 2020
Glycodendrimers	structurally and functionally mimic natural polysaccharides, glycoproteins and mucins based dendrimers, targets carbohydrate specific receptors	Biocompatibility, non-toxicity, effective and strong biniding to lectin, cell specific targeting	Stability problems	Inhibit adhesion of HIV (human immune-deficiency virus), inhibit chlora toxin, inhibit bindling of influenza virus and *E. Coli* bacteria such as *Streptococcus* suis, *Pseudomonas aeruginosa*, vaccines and drug delivery vehicles	Zhang et al. (2022), Arabi et al., 2020, Roy et al. (2013), Gillies (2011)
Triazine Dendrimers	Structure with Orthogonal functional end groups for surface modification possibility, scalable	synthetic versatility, well-defined structure, orthogonal functional group provides space for drugs and ligands attachment	solubility limitations, intrinsic toxicity	Cancer drugs (paclitaxel, camptothecin) brefeldin A (antiviral) and desferrioxamine), non-viral DNA and RNA delivery systems, in sensing applications, and as bioactive materials	Haiba et al. (2022), Apartsin et al. (2022), Lim et al., 2019
Polyglycerol Dendrimers	Hyperbranched polymeric structures with multiple peripheral hydroxyl groups provides attachment point to other groups for surface modification	excellent water-solubility, non-toxicity, and minimal non-specific interactions in biological environments, good biocompatibility, low polydispersity	Tedious multiple step synthesis process and purification problems	drug delivery, gene transfection, biomedical imaging, and diagnostics	Maysinger et al. (2020), Sharma et al. (2020), Jiang et al. (2016), Ooya and Lee 2022
Citric acid dendrimers	Highly branched, monodisperse, stable molecular level, low polydispersity, micellar structure	Water soluble, biocompatible, less toxic	Interaction with biological membranes cause membrane disruption *via* nanohole formation, membrane thinning and erosion	Drug/gene delivery systems	Namazi et al. (2017), Nangare et al. (2021)
Polyether dendrimers	spherical, highly branched, functional dendrimers with surfaced positively charged ether groups	Low polydispersity, high surface area to volume ratio, low viscosity, high solubility and miscibility and adhesiveness	Small yield, toxicity	Biomedical and tissue engineering applications	Knauer et al. (2022), Dhanikula et al. (2008), Michael et al., 2001

Fibrin-binding CREKA, small pentapeptide of Cys-Arg-Glu-Lys-Ala specifically binds to fibrin; p42-MAPK siRNA, p42-Mitogen-activated protein kinases siRNA KLAK, Lysine-Leucine-Alanine-Lysine; Bcl- B-cell lymphoma 2; VEGF siRNA, Vascular endothelial growth factor anti-GFP siRNA- anti-green fluroscent protein siRNA; TRAIL, tumor-necrosis factor related apoptosis-inducing ligand.

### 6.1 PAMAM dendrimers

**FIGURE 3:**
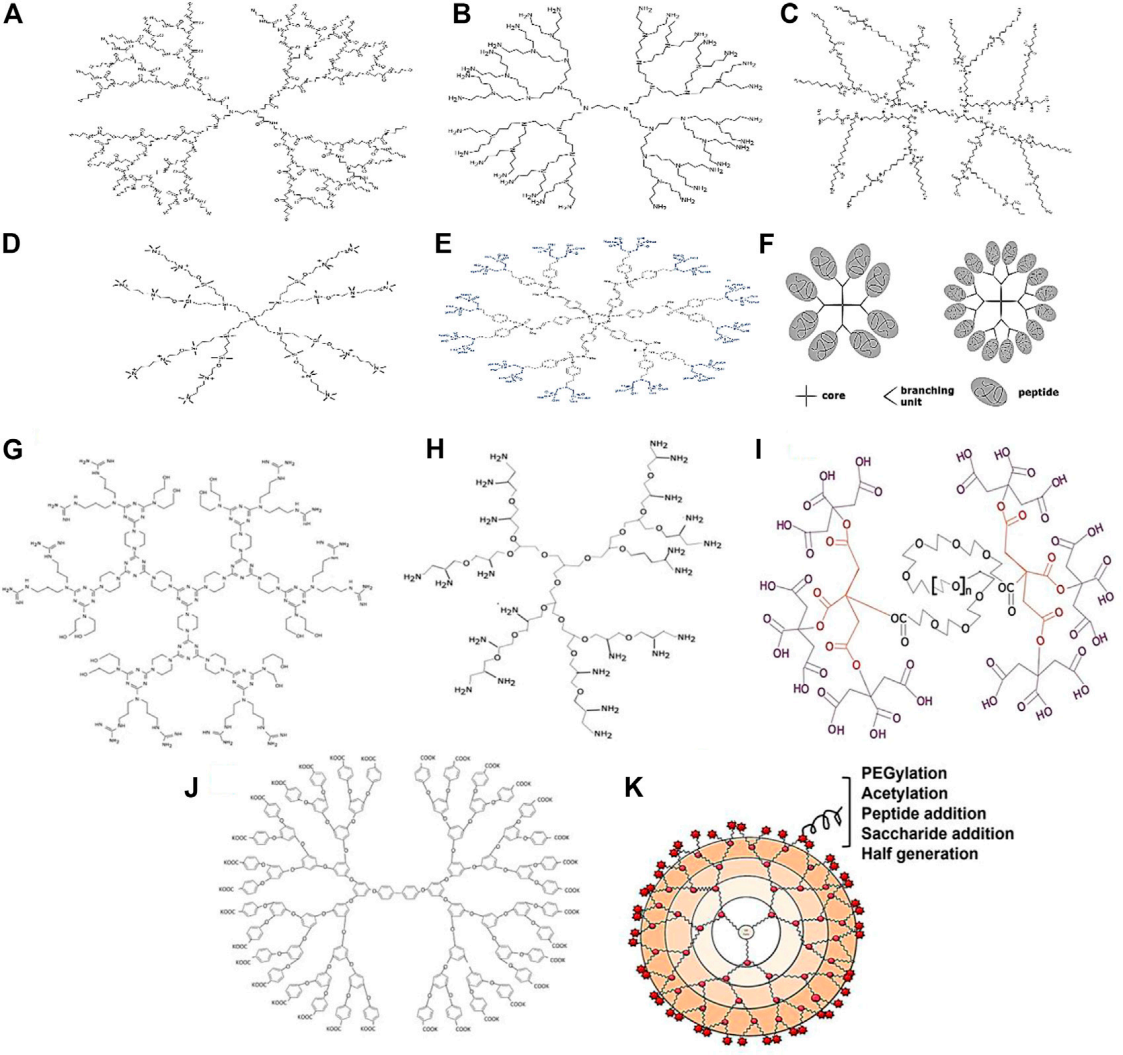
Types of dendrimers: **(A)** PAMAM Dendrimers; **(B)** PPI Dendrimers; **(C)** PLL Dendrimers; **(D)** Carbosilane Dendrimers; **(E)** Phosphorus Dendrimers; **(F)** Peptide dendrimers. Types of dendrimers: **(G)** Triazine dendrimers; **(H)** Polyglycerol dendrimers; **(I)** Citric acid dendrimer; **(J)** Polyether dendrimers; **(K)** Surface engineered dendrimers.

Polyamidoamine dendrimers essentially have a central ethylenediamine core with ascending divisions with amide groups which form a wall of the spherical structure and at the surface of each terminal with amine functional groups (Figure 3A). PAMAM dendrimers are good candidates for carrying drugs and peptides because they are biocompatible and have well-defined spherical nanoparticles with bifunctional modifying parts. Dendrimers PAM are used in the nanomedicine platform to maximize the bioavailability of drugs and minimize the number of doses required (Pedziwiatr-Werbicka et al., 2019). However, there are some limitations associated with the use of PAMAM, particularly those with positively charged groups. It leads to the accumulation produces cytotoxicity. PAMAM dendrimers of G5 are mostly considered as a non-toxic (Beezer et al., 2003). To address the aforementioned limitation, the surface is reconfigured with polyethylene glycol (PEG). Compared to “other dendrimers,” PAMAM has the broadest range of applications in therapeutics such as antibacterial, antiviral, antioxidant, and diagnostic agents.

### 6.2 Polypropylenimine dendrimers

The dendrimer polypropylenimine (PPI) is the first known dendrimer used industrially as a therapeutic agent. There are also other names used for PPI dendrimers which are astramol or butylenediamine (BDA) or “polypropyleneamine” (POPAM) (Noske et al., 2020). These dendrimers mainly consist of butylenediamine as the core and repeat propylenimine branching units *via* sequential Figure 3B is the reaction of Michael’s addition of acrylonitrile to a primary amino group, followed by hydrogenation of the nitrile groups to form primary amino groups (Sherje et al., 2018). The amino-terminal groups contribute to its solubility in water. PPI Dendrimers are characterized by their ability to improve the water solubility of hydrophobic drugs entrapped in the hydrophobic inner crevices of PPI due to this property. Cell membranes can be destabilized by positively charged PPIs, leading to cell lysis. As a result, PPIs have a lower ability to load drugs than PAMAMs. Furthermore, the PPI/drug complex has lower stability than PPI alone. PEGylation and acetylation of the surface groups are selected. Acetylation is preferred as it is extremely efficient and able to penetrate deeply. Moreover, the steric hindrance of the PEG chain can affect the interaction of the entrapped drug molecules with the surface functional groups (Fant et al., 2010). Diaminoethane or diamino-propane would form the central functional groups in Poly (ethyleneimine) (PEI) dendrimers, a subclass of PPI dendrimers.

### 6.3 Poly-l-lysine dendrimers

Dendrimers containing poly-l-lysine residues such as poly-l-lysine dendrimers (PLL) or dendrigraft poly-l-lysine dendrimers (DGL) also possess lysine residues (Figure 3C). These dendrimers have higher cytocompatibility, lower cytotoxicity, convenient enzymatic degradation and resultant efflux of low molecular products (Ryan et al., 2013). Furthermore, DGL dendrimers have been researched for the use of delivery vectors for siRNAs. “The ability to respond to stimuli on demand by incorporating specific protein sequences into PLL dendrimers is quite tantalizing”. Higher-generation PLL dendrimers have exhibited improved gene transfection efficiency whereas conventional linear poly (lysine) dendrimers have exhibited lower genome transfection efficiency and higher cytotoxicity compared to PLL dendrimers (Byrne et al., 2013).

### 6.4 Carbosilane dendrimers

Carbosilane dendrimers consist of carbon and silicon molecules as building blocks. With the widespread use “of silicon chemistry, a new class of carbosilane dendrimers with hydrophobic scaffolds and superior thermal stability have emerged (Figure 3D) (Uchida et al., 1990). The chemical properties of silicon are utilized in dendrimers preparation because they allow the “nucleophilic molecules to reach the electrophilic silicon (Si+) easily” (Zhou and Roovers, 1993). However, the presence of the C-Si bond in carbosilane dendrimers provides low polarity and high energy which makes them more hydrophobic compared to other dendrimers (Sepúlveda-Crespo et al., 2015). Although they have a hydrophobic endoskeleton, carbosilane dendrimers can be modified to polar molecules by surface chemistry with polar moieties such as Si-H, Si-Cl, Si- CH = CH2, and Si-CH2CH = CH2, allowing the introduction of various other intriguing inorganic, organic, and organometallic substituents. This would lead to increased applications in the pharmaceutical fields. The ability of the generation 2 ammonium-terminating carbosilane dendrimer “has been tested on a variety of cell types including the glial cells, progenitor cells, leukocytes, granulocytes and human peripheral blood mononuclear cells, general pluripotent stem cells, primary cells as well as suspension cells” (Liu et al., 2012).

### 6.5 Phosphorus dendrimers

Cationic dendrimers are dendrimers with cationic phosphorus as the core and decorated surface groups that have been studied in a variety of biological and theragnostic applications due to their special properties (Figure 3E). In phosphorus dendrimers, the “presence of phosphorus at each branch point” and reactive end groups provide a hydrophilic shell and hydrophobic backbone that can affect their internalization into cells. Moreover, they have effects on the growth of cells as well such as neurons, immune and cancer cells (Caminade and Majoral, 2013). Rolland et al. (2008) investigated on phosphorus-terminated dendrimers that could be explored for immunotherapy to target and activate monocytes. Based on the study conducted, such dendritic materials have been shown to be able to affect the aggregation of amyloid oligomers and tau protein in neurodegenerative diseases (Wasiak et al., 2012). Polyphosphorhydrazone (PPH) dendrimers have also shown the ability to deliver antisense siRNAs to target cells (Dzmitruk et al., 2015) and treat HIV infection with gene therapy (Briz et al., 2012).

### 6.6 Peptide dendrimers

Peptide “dendrimers are macromolecules which comprise either branched polypeptide core” or radically arranged peripheral polypeptide chain or both (Figure 3F) (Sadler and Tam, 2002). These dendrimers are divided into three categories. The first category is grafted dendrimers with amino acids chain only at their surface. The second category is grafted dendrimers composed entirely of amino acids, while the third category consists of amino acids that branch both in the core and on the surface and have non-peptide branching units. Divergent and convergent techniques are often used to synthesize peptide dendrimers, and with the advent of solid-phase peptide synthesis methods, extensive “libraries of peptide dendrimers can be prepared and tested for desired properties.” Peptide dendrimers are used as surfactants as well as multiple antigen peptides (MAP) (Bruckdorfer et al., 2004), protein analogs (Thompson and Scholz, 2021), drugs, and gene transporters in the biomedical field. They are also used “as contrast agents for magnetic resonance imaging and angiography, fluorescence imaging” and in serum analysis (Choi et al., 2000).

### 6.7 Glycodendrimers

Dendrimers that comprise carbohydrates “moieties such as monosaccharides (glucose, mannose, galactose) (Woller and Cloninger, 2001) and disaccharides (chondroitin sulfate) (Roy and Baek, 2002) ”into their structure are referred as glycodendrimers. Although most of “the researched glycodendrimers have sugar residues on their exterior surfaces, glycodendrimers” with a sugar unit as the central core in which all branches would emerge have been discovered as well. In general, glycodendrimers are classified into three types: “Carbohydrate-centered, carbohydrate-based and carbohydrate-coated dendrimers (Oliveira et al., 2010). There is a potential application for these dendrimers which is site-specific delivery to lectin-rich” tissues. These dendrimers are predicted to have a stronger affinity for lectin-anchored systems than monosaccharide-anchored systems (Mousavifar and Roy, 2021).

### 6.8 Triazine dendrimers

Triazine dendrimers are aptly named as they “consist of 1,3,5-triazine rings as branches with amine groups at both ends” (Figure 3G). A study was conducted recently on the siRNA transport ability of a series of “triazine dendrimers with different core structures, generation numbers and surface functions”. Dendrimers with inflexible structures and “arginine-like or hydrophobic terminals had the most efficient siRNA gene repression effects” on Hela cells in an experimental luciferase model (Simanek, 2021). Triazine dendrimers are deemed suitable for various biomedical applications due to their liquid crystalline structure and non-linear optical properties. In one study, triazine dendrimers were evaluated for their potential for drug delivery. The results showed that they are suitable for hydrophobic drugs as solvents and carrier systems and are not toxic to organs at a dose of up to 10 mg/kg *via* the intraperitoneal route in an animal model. However, these dendrimers should be explored further for various applications with or without surface functionalization by appropriate moieties (Merkel et al., 2010).

### 6.9 Polyglycerol dendrimers

Dendritic polyglycerols are hyperbranched structures that are made of glycerol with a wide range of sizes (at the nanoscale) and functional groups at their ends which allow them to interact with different biological receptors (Maršić et al., 2021). Sheikhi Mehrabadi et al. (2015) have developed polyglycerol dendrimers (PG) with a variety of cationic amine end groups, including a star-shaped oligoamine shell (Figure 3H). These dendrimers have a neutral biocompatible aliphatic polyether core, numerous siRNA-binding and complexing amine end groups. PG-PEHA (Polyglycerol-pentaethylene hexamine) was the most efficient at silencing genes, whereas polyglycerol-amine (PG-NH2) was the least effective. “The favorable primary amines at the 1,2-position of PG-NH2” might be the reason for the reported high efficiency in the transfer of siRNA (Sheikhi Mehrabadi et al., 2015). Moreover, PG-NH2 could be used to deliver siRNA in animal models by intravenous injection, resulting in efficient gene silencing with low toxicity. This further indicates the potential utility of the dendrimer for siRNA therapy delivery (Sheikhi Mehrabadi et al., 2015).

### 6.10 Citric acid dendrimers

Citric acid dendrimers can be good candidates for an efficient drug delivery system as they are relatively stable in water with good drug deposition and release properties (Figure 3I). Namazi et al. prepared β-cyclodextrin (β-CD)-modified citric acid dendrimers with -cyclodextrin (CD) to increase the loading capacity and encapsulation properties of the dendrimers. The results showed that by increasing the number of branches, it had further increased the internal cavity, hence responsible for the resulting loading potential (Namazi and Hamrahloo, 2011). Similarly, Namazi and his colleagues prepared dendrimers with PEG as the central core and repeating citric acid units as surface functionalized groups (Namazi et al., 2011). In the viability assay, the viability of HT1080 cells which were exposed to the PEG-citric acid dendrimer was found to be more than 80% up to 2 days at a high concentration of 1 mg mL-1. In other toxicity tests, the dendrimers were shown to be extremely safe. The tests done included hemolysis assay, lactate dehydrogenase assay and prothrombin time assay (Naeini et al., 2010). The biocompatible dendrimer exhibited high drug loading and prolonged drug release, indicating its potential application as a vehicle for cancer treatment (Adeli et al., 2013).

### 6.11 Polyether dendrimers

Polyether dendrimers are functional dendrimers that are spherical and highly branched with cationic ether groups on the outer surface. Polyether dendrimers were synthesized for the first time by Hawker and Frechet in 1990 *via* the convergent approach (Figure 3J) (Hawker and Frechet, 1990). This polyether group is composed of the core material “1,1,1-tris(4′-hydroxyphenyl) ethane and the branching material benzyl bromide and 3,5-dihydroxybenzyl alcohol”. Jayaraman et al. described the synthesis of dendrimers with a backbone that consists of aliphatic polyethers. Due to the presence of “2-hydroxymethyl-1,3-propanediol moieties”, the prepared dendrimers emerged as good prospects for drug delivery with improved solubility (Kumbhar et al., 2021).

In another study, Malik et al. performed *in vitro* tests on the biocompatibility of these convergently synthesized polyether dendrimers. The findings indicated that such dendrimers with carboxylate and malonate interfacial conjugates were more biocompatible and hemolytic than cationic dendrimers. However, they were not hemolytic up to 1 h but were as hemolytic as anionic dendrimers after 24 h. It seems that these biodegradable polymers may less hazardous than standard dendrimers and their usage for drug delivery could be extended (Duncan, 2014).

### 6.12 Surface tailored dendrimers

It seems that making changes to the surface is one of the most effective ways to make dendrimers less harmful (Figure 3K). In this approach, the exposure of cationic groups such as amino groups to the surface of the dendrimer is minimized by modification or decoration with natural or anionic molecules to prevent their electrostatic interaction with cell membrane molecules, thus avoiding cytotoxicity mediated by cationic groups. Apart from reduced toxicity, surface-engineered dendrimers also can improve drug loading, biodistribution, pharmacokinetic profile, solubility, site-specific targeting stability, antimicrobial activity and gene delivery efficiency (Satija et al., 2007). There are several surface engineering approaches to modify surface groups on dendrimers such as PEGylation through the addition of PEG, which increases drug loading and decreases hemolytic toxicity, as seen in PAMAM dendrimers (Santos et al., 2019), saccharide addition *via* maltose, which decreases hemolytic toxicity, as seen in PPI dendrimers (Bhadra et al., 2003), acetylation by adding an acetyl group lessening toxicity effects and increase absorptivity of PAMAM dendrimers (Klajnert et al., 2008), bisection by adding carboxylic acid to minimize cytotoxicity (Kolhatkar et al., 2007) and peptide conjugation by tripeptide (arginine-glycerol aspartate) to minimize toxicity of cationic dendrimers (Jevprasesphant et al., 2003).

## 7 Brain tumor drug targeting approaches

The main obstacles in the detection and treatment of cancer are the development of drug/gene delivery systems that selectively target cancer cells while leaving normal healthy cells/tissues unaffected. This could be achieved by effective delivery of anticancer drugs into tumor cells (Khizar et al., 2021). Nanotechnology-based delivery systems, namely, phytosomes, liposomes, nanoparticles, carbon nanotubes, neosomes, and dendrimers, are believed to be the most practical choices. The nanocarriers produced must overcome a number of physiological and biological obstacles. Their use as delivery systems requires that their size, biocompatibility, and surface functionality are suitable to promote the binding of specific drugs and diagnostics to their target sites and avoid unwanted interactions (Truskewycz et al., 2022).

**FIGURE 4:**
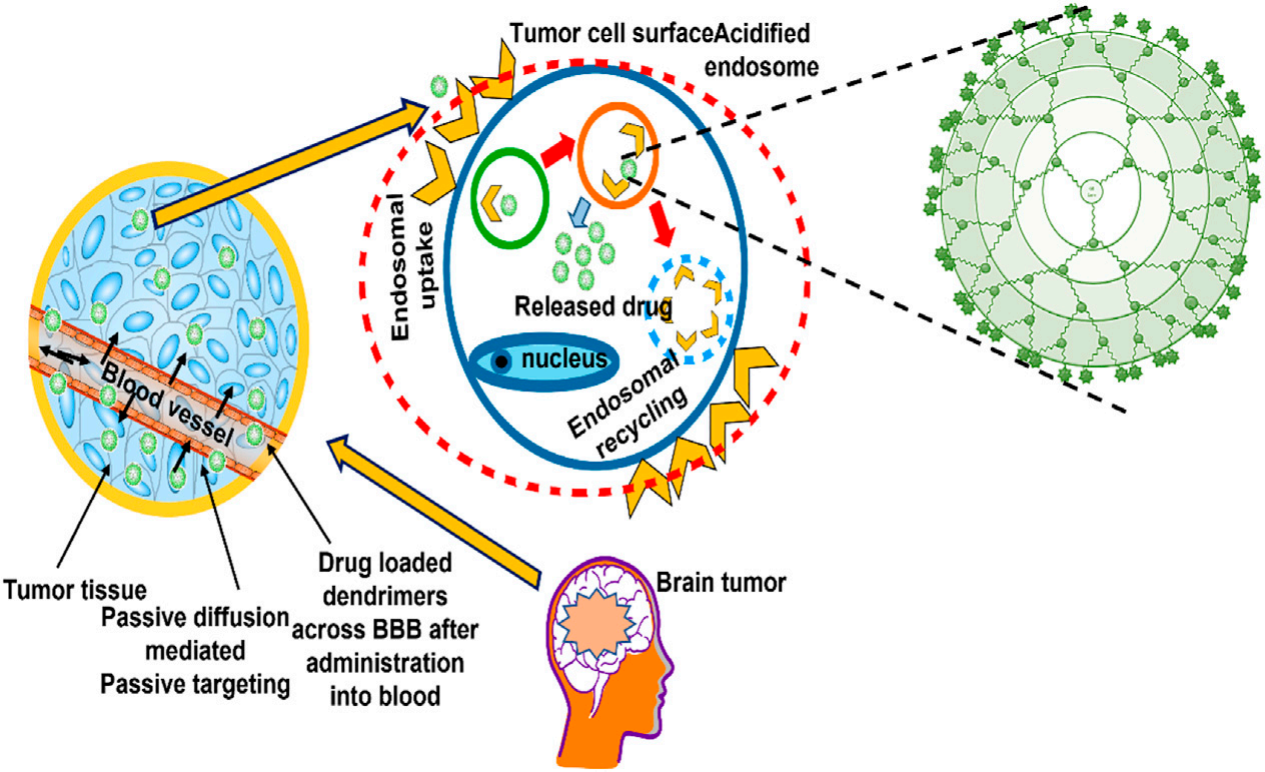
Drug targeting approaches (active and passive) mediated by dendrimers in brain tumor.

Nanocarrier systems can be employed to transport therapeutic agents or genetic material into cancer cells or tissues. This can be done either actively or passively (Attia et al., 2019). Passive targeting enhances the amount of drug or the delivery of a drug to a specific region due to its physicochemical characteristics (particle size, circulation time), pathophysiological conditions (hypoxia, inflammation) and tumor biology (leakiness, vascularity) (Iyer et al., 2014). Active targeting, on the other hand, requires specific adjustments to the delivery system that would result in the coupling of active agonists with significant selectivity for a specific cell or organ in the body (Figure 4).

### 7.1 Passive targeting approach

The use of polymeric delivery systems takes advantage of a drug’s pharmacokinetic properties, such as its solubility, half-life, and prolongation of plasma circulation time, to achieve optimal passive targeting. This ensures that the drug matrix is passively transported to the solid tumour (Ekladious et al., 2019). Furthermore, passive targeting certainly depends strongly on two parameters: The tumour endothelial permeability to macromolecules and the presence of reduced lymphatic outflow. These two factors determine how well passive targeting works. When these factors are improved, the likelihood of passive targeting would increase. The enhanced effect of permeation and retention, often called as the EPR effect, is a phenomenon unique to tumours. It was first discovered and stated by Matsumura and Maeda in 1986. Because tumours have defective blood vessels, they form vascular permeability factors. These factors ensure that the tumour tissue receives adequate nutrients and oxygen, allowing the tumour to develop rapidly. Aliphatic polyester dendrimers containing dimethylolpropionic acid have been identified as a promising possibility for the preparation of therapeutic anticancer conjugates. The results of *in vitro* and *in vivo* evaluation showed that the water-soluble polyester dendrimers were biocompatible. The delayed aggregation of dendrimers in important organs results in a prolonged period of dendrimer-mediated drug administration, which appears to be beneficial for the EPR effect of passively targeting tumours (Kheraldine et al., 2021). In addition, tumour cells would stimulate dilatation of blood channels through excessive release of permeability mediators (Greish et al., 2003; Gillies and Fréchet, 2005).

Polymeric drug conjugates and drug loaded polymeric micelles, dendrimers, polymeric nanoparticles and carbon nanotubes would selectively accumulate inside solid tumor owing to the EPR effect (Puri et al., 2009; Shcharbina et al., 2013). Constructions with hydrophilic surfaces and molecular weighing over 25 kDa–30 kDa would provide enhanced chances of targeting tumors by virtue of EPR, which is accompanied by a longer retention time during circulation (Kesharwani et al., 2015). Etrych et al. (2008) developed a biodegradable PAMAM dendrimer with a semi-telechelic Hydroxypropyl Methyl Cellulose (HPMA) polymer embedded with doxorubicin as the core for passive tumor targeting. The findings revealed that the dendronized nanoparticles with diameters ranging from 10 to several hundred nanometers were able to achieve passive targeting through the EPR effect (Etrych et al., 2011). The size range of nanocarriers will determine whether or not they can be retained and localised in target areas. There is general agreement that dendrimers up to 10 nm–20 nm in diameter are most appropriate for passive tumour targeting (She et al., 2013).

Massive membrane proteins and other macromolecules which are coupled with dendrimers would decrease their plasma clearance, resulting in increased half-life in the bloodstream. As a result, these conjugation systems provide a prolonged and precise delivery to tumor targets. Furthermore, a cisplatin-loaded 3.5 G PAMAM dendrimer demonstrated a 50-times rise in cisplatin accumulation at the tumor position when compared to free drugs (Kaminskas et al., 2011).

### 7.2 Active targeting approaches

Chemotherapy is an essential procedure for cancer treatment in modern times but there is a lack of antineoplastic drugs that can act against the tumor mass. To find solutions to these problems, researchers and scientists have focused their efforts on developing novel anticancer drugs and drug delivery systems. Some examples of these innovations include tailored drug delivery methods that have high therapeutic efficacy and low toxicity. Active targeting is a strategy that can decrease the absorption of a drug in normal tissue and increase its concentration in malignant tissue (Janaszewska et al., 2019). It was found that the most efficient method for drug accumulation in solid tumours is known as EPR, which is achieved by passive tumour targeting. Although the majority of the pharmaceuticals are notoriously non-diffusible, intracellular targeting of cancer cells *via* passive diffusion of these substances is notoriously hard. In addition, administration of a low dose of drugs to certain cancer cells can lead to ineffectiveness of chemotherapy or other negative outcomes, such as the growth of cancers resistant to multiple drugs (also known as MDR malignancies) (Basile et al., 2012). Passive targeting and the EPR are therefore only able to deliver drugs to solid tumours that are porous and permeable. On the other hand, the EPR effect does not occur in a number of malignant tumours that are resistant to radiation because these cancers are impermeable (Gottesman et al., 2002). Active targeting overcomes some of these limitations by attaching precisely targeted ligands to the outside of nanostructures that exert an attractive force on specific receptors on cancer cells (Gottesman et al., 2002).

Dendrimers have several polar functional groups which might attached to a diversity of ligands on their surface to target tumors in active targeting. In one study, polymethacrylate (PMA)-crosslinked PAMAM dendrimers were prepared so that medications could more easily targeted the acidic microenvironment of the tumour. Folate-PEGylated PMA-PAMAM had a significant effect on drug accumulation and tumor regression at the tumor site (Shen et al., 2012).

The adhesion molecule integrin αvβ3 is commonly expressed in prostate, breast, ovarian, glioblastoma and melanoma cells. Peptides that include the amino acid sequence Arg-Gly-Asp (RGD) show a better affinity for the integrin αvβ3 protein (Li and Xu, 2005). PEGylated PAMAM dendrimers coated with RGD embedded with Doxorubicin (DOX) prolonged plasma circulation time, drug accumulation and bioavailability in brain tumors compared to DOX solution alone (Zhang et al., 2011).

According to the findings of Gupta and colleagues, a dendrimer loaded with DOX and folate-conjugated PPI was the improved option for targeting cancer. The novel dosage showed improved drug stability, release profile and toxicity, as well as better drug uptake in the cancer cell MCF -7 (Gupta et al., 2010). There are several ligands for targeting purposes in the brain and other body tissues such as thiamine, glucose, choline, serum albumin, folate, lactoferrin, L-glutamate, L-aspartate, folic acid, nucleoside, biotin, oligopeptide and aptamers. Nanoparticles that can be formed on the surface of dendrimers efficiently controlled the tumor progression (Lockman et al., 2003; Huang et al., 2008; Ulbrich et al., 2011).

Across the brain many transporters such as glucose transporter 1 (GLUT1), vitamin C transporter 2 (SVCT2), Na + -dependent vitamin transporter (SMVT), L-amino acid transporter 1 (LAT1), mono-carboxylic acid transporter 1 (MCT1) etc., works as carrier mediated transporters to transport nutrient through the BBB. Modification of surface characteristics of Dendrimers with the substrates or their analoues can promotes drug into brain *via* mentioned transporters ((Zhao et al., 2015; Jiang et al., 2021; Zhao et al., 2021).

Another most widely used way to internalized large drug moieties and growth factors in brain through brain tumor targeted delivery systems is receptor mediated transport. Most commonly expressed receptor on brain are low density lipoprotein receptor (LDL-R), apolipoprotein E (ApoE) receptor, EGFR, transferrin receptor (TfR), insulin receptor (IR) and integrin receptor (αvβ3) and can be employed efficiently to promote drugs and diagnostic agents into the brain for brain tumor treatment and imaging respectively (Yeini et al., 2021).

## 8 Application of dendrimers as drug delivery systems for the brain

With the help of dendrimers, nucleic acids and drugs can be sent to the brain and cancer cells without using a virus. This is due to the high-branched structure and the available internal cavities of these polymers which make them excellent delivery systems for genes and drugs.

### 8.1 PAMAM dendrimers as brain drug delivery

The BBB functions as a filter to control molecules that have entered the brain from the blood (Li et al., 2020). A series of specialized cells and transporters have been developed to manage the chemical environment of the CNS. It consists of endothelial cells that form specialized capillaries and extravascular components such as astrocytes, pericytes, and interneurons that make up the BBB (Xu et al., 2014). CNS nutrients and waste are regulated by these components which constitute as a physical barrier. The extracellular nucleases and peptidases, intracellular monoamine oxidase and cytochrome P450 enzymes are some of the proteins and enzymes that prevent toxicity of the central nervous system (Pisoschi et al., 2021).

The potent anticancer drug methotrexate is an antimetabolite of folate that has been shown to be effective in the treatment of various cancers. PAMAM dendrimers do possess capability of good drug carriers owing to precise description of their structures and a variety of different types of groups (De et al., 2022). Wu and colleagues utilized methotrexate to develop a drug carrier that targeted the epidermal growth factor receptor (EGFR) as well as their mutant isoform EG-FRvIII. This was done by binding an average of 12.6 methotrexate molecules to every fifth PAMAM dendrimer molecule, which was then conjugated to cetuximab. According to the researchers, particular molecular targeting is just one of several properties that an antibody-drug bioconjugate must meet to be therapeutically useful. However, no therapeutic benefit was observed when tumor mice were administered the bio-conjugate *via* CED instead of free methotrexate or cetuximab (Wu et al., 2006).

Table 2 presents an overview of the numerous PAMAM dendrimer types that have been utilized.

**TABLE 2:** Different polymer-based dendrimer generation and results of the study.

Dendrimer type	Drug used	Result of study	References
Poly (amidoamine)	Paclitaxel	Developed FA-PMA-PAMAM dendrimers that are pH sensitive and release the drug acidic tumor microenvironment *via* active and passive targeting	Shen et al. (2012)
	DOX	Dendrimer complexes would be able to deliver a significantly higher amount of drugs to brain tumors, resulting in more successful therapeutic outcomes	Li and Xu (2005)
	Docetaxel	Hybrid dendrimer that can cause toxicity in U87MGMG glioblastoma cell line but non-toxic on control cells	Zhang et al. (2011)
	paclitaxel	Improve the penetration of paclitaxel 12 times greater through brain endothelial cells	Gupta et al. (2010)
	Apoptin	Phenylalanine, histidine and arginine decorated PAMAM dendrimer loaded with apoptin that induce apoptosis in primary glioma cell lines	Lockman et al. (2003)
	Emcyt and Podofilox	In order to increase the efficacy of D-PODO and D-EM, the sequence of the release which would lead to sustained action	Ulbrich et al. (2011)
	Pentapeptide	PAMAM modified with CREKA penetrate deeply into GBM tissues and enhance retention	Huang et al. (2008)
	Peripheral-type benzodiazepine receptor (PBR)	TSPO-targeted G (4)-PAMAM-FITC dendrimer targets the mitochondria of gliomas 6 cells and quickly taken up by the endocytosis pathway	Xu et al. (2014)
	RNA (siRNA) and Adriamycin	Dendrimers can be used to administer drugs and siRNA to cancer cells that are resistant to anticancer drugs	(Abbott et al., 2010)
	Doxorubicin	Poly (2-methacryloyloxyethyl phosphorylcholine) modified G3-PAMAM dendrimer enhanced tumor targeting in U-87 tumor mouse model with reduced toxicity	Ban et al. (2021)
	Doxorubicin	iRGD-modified G4 PAMAM dendrimer *via* enhanced permeability increases DOX accumulation in tumor and decreases tumor diameter	Wang et al. (2014)
	Doxorubicin	Folic acid conjugated borneol modified G-5 PAMAM dendrimers increase accumulation of DOX in C6 glioma xenograft rat model with prolonged survival time	Xu et al. (2016)
	Cy5-NHS ester	Galactose, mannose and glucose decrorated PAMAM dendrimers significantly enhance tumor-associated macrophages and microglia targeting *via* increasing brain penetration and cellular internalization	Sharma et al. (2021)
Carbosilane	siRNA	Crossing the BBB without cytotoxicity	(Kim et al., 2018)
	SiRNA (HIV-1 Nef silencing)	Gene silencing in human astrocytes without causing toxicity	Serramía et al. (2015)
Poly (L-lysine)	HSV-TK and angiopep-2	High transfection efficiency, good biodegradability, anti-glioma effect and increase survival chances in human GBM animal model	Igartúa et al. (2018)
	TRAIL and HSV-TK	PPL-PEI combination therapy provides cost-effective treatment in GBM treatment	Swami et al. (2015)
	Curcumin	A Poly (L-lysine) dendrimer complex that can be used to enhance RNAi therapeutics and nanomedicine for brain tumors	(Braun et al., 2005)
	Minocycline	Neuroinflammation is reduced at lower doses	(Dufes et al., 2005)
Poly (propyleneimine)	Doxylamine and Sodium deoxycholic acid	Reduced cytotoxicity and sustained drug release with formulations that increase drug loading capacity	Shen et al. (2012)
	β-Galactosidase and Hepcidin	A gene delivery system that utilizes a polypropylenimine dendrimer bearing transferrin which is highly promising	Demeule et al. (2008)
Peptide dendrimers	N^1^-butyl and N^1^-aminopentane tryptophan Peptide dendrimers	Terminal functionalized (N^1^-butyl and N^1^-aminopentane tryptophan) amphiphilic peptide dendrimers have high ROS scavenging activity with GBM cells killing potential and 85–95% retaining of viable astrocytes in brain	Sowińska et al. (2022)
	Histidine, nitro-arginine or proline functionalized ornithine dendrimers	Dendrimers have targeted cytotoxicity on glioblastoma cell lines U87 and T98G	Cieślak et al. (2020)
Triazine-Phosphorus dendrimers	copper (II) or gold (III) complexes and branched hydrophobic fragment bearing dendrimers	Showed higher cytotoxicity on glioblastoma stem cells (BTSC233, JHH520, NCH644, and SF188 as compared with temozolomide	Apartsin et al. (2022)
	phosphorus dendron-micelles	Showed moderate anti-proliferative activity in glioblastoma cell lines U87	Qiu et al. (2021)
Polyether-copolyester dendrimers	MTX-loaded glucosylated and non-glucosylated dendrimers	Glycosylated MTX polyether-polyester dendrimers showed good permeability in BBB and endocytosed effectively in U87 MG and U 343 MGa cells	Dhanikula et al. (2008)
Amphiphilic glycodendrimers	Terminally functionalized with mannose and glucose	Showed effective cellular uptake in microglia and astrocytes and cancer cells	Zhang et a.,2022

FA-PMA-PAMAM, Folic acid conjugated polymethacrylate polyamidoamineD-PODO and D-EM- PAMAM dendrimer loaded with natural podophyllotoxin and estramustine respectively; TSPO-targeted G (4)-PAMAM-FITC; translocator protein targeted PAMAM- Fluorescein isothiocyanate; HSV-TK, herpes simplex virus thymidine kinase; PPL-PEI, poly(propylene imine- polyethyleneimine.

### 8.2 PAMAM dendrimers in small molecules brain delivery

PAMAM dendrimers could be used to carry peptides and drugs to specific sites in a way that is effective and efficient (Abedi-Gaballu, et al., 2018). This is because they can enhance the biocompatibility of active compounds and decrease the number of times they need to be taken. The fact that PAMAM dendrimers produced natural podophyllotoxine and estramustine more bioavailable showed that they could be used as carriers. Along with stopping and killing more cells, PAMAM dendrimers also modified the manner in which these antimitotic agents were released. It also made these antimitotic agents work better at stopping tubulin polymerization, which is important for glioma cell survival (Tarach P & Janaszewska, 2021; Dixit & Sen, 2013).

In one work, PEGylated dendrimers, wheat germ agglutinin, and transferring ligands were used to encapsulate doxorubicin in G4 PAMAM dendrimers. The ability of the dendrimers to cross the BBB was enhanced by targeting wheat germ agglutinin (WGA) and transferrin (Tf). In addition, microscopic study showed that the nanoparticles had a size of about 20 nm. Compared with free drugs and dendrimers without Tf and WGA, the dendrimers containing Tf and WGA delivered a higher payload of doxorubicin to brain tumour sites. In another study, Swami and colleagues utilized PAMAM dendrimers to combine docetaxel (DTX) with p-hydroxylbenzoic acid (pHBA). The findings suggested that pHBA has a strong affinity for beta receptors which are found primarily in the CNS. Compared to free Docetaxel (DTX), dendrimers containing G4-pHBA-DTX delivered more drugs to the brain (Swami et al., 2015; Florendo et al., 2018; Igartúa et al., 2018; Vasconcelos-Ferreira et al., 2022).

### 8.3 PAMAM dendrimers in brain delivery of genes

When it comes to how they interact with nucleic acids, small molecule drugs are the complete opposite of dendrimers. Nucleic acids (DNA or RNA) can be delivered using dendrimers with surface amines. During physiological conditions, dendrimers are formed when amines interact with nucleic acids to produce dendrimer complexes. In terms of their physicochemical properties, hydroxyl and carboxyl groups are considered neutral under physiological conditions, whereas nucleic acids are considered to be anionic. Therefore, dendrimers containing these surface functional molecules cannot bind nucleic acids. The dendrimers and nucleic acids that form on amine-terminated dendrimers can exhibit considerable variation in size and structure due to the multiple charges in both (Dey et al., 2022). In addition to individual characterizing dendrimers (generations), the sizes are affected by the nucleic acid, the N/P ratio (also known as the charge ratio) between dendrimers and nucleic acids and the solvent properties (Sahu et al., 2023). As the dendrimers are being generated, their DNA binding affinity would increase. At charge ratios close to 1, the complexes would typically aggregate and fail. Therefore, dendrimer complexes with a higher density tend to have a smaller size distribution and better transfection efficiency. Apart from that, complexes with an increased dendrimer generation ratio have an increased charge ratio as well (two times more amines than phosphates in a dendrimer complex). Generally, nanometer-sized dendriplexes are formed when the generations and the N/P ratio are higher (Demeule et al., 2008; Huang et al., 2008). The efficiency of transfection varies greatly between dendrimer-nucleic acid complexes. In addition to depending on the structure of the complex, it also varies based on the type of cell and the density of the cell population. Dendriplexes have shown promising results in the field of biology. Therefore, it is an excellent opportunity for researchers to apply a range of analytical techniques to accurately describe the complexes formed by dendriplex formation.

According to a study done by Huang and his team, transferrin that is attached to dendrimer DNA dendriplexes of PAMAM increased the number of genes that were expressed in the brain twice. The same study also revealed “that conjugating lactoferrin to PAMAM dendrimers” using PEG spacers boosted dendrimer brain absorption by “4.6-fold compared to non-conjugated PAMAM dendrimers and by 2.2-fold compared to dendrimers conjugated to transferrin” in BALB/c mice (Huang et al., 2008). Low density lipoprotein (LRP) receptors are extensively produced in mammalian neural cells. Angiopep has demonstrated selectivity in targeting LRP receptors. “Angiopep-PEG-PAMAM loaded with DNA” caused higher gene expression “in the cortex, caudate putamen, hippocampus and substantia nigra of BALB/c mice than unconjugated PAMAM loaded with DNA (Ke et al., 2009).

### 8.4 Carbosilane dendrimers as a brain drug delivery

Researchers have addressed the possibility that dendrimers may treat or ward off neurological and degenerative diseases of the brain. Because of the BBB, treating and/or preventing diseases that affect the central nervous system can be extremely difficult. One of the factors contributing to the ineffectiveness of this barrier is inadequate drug penetration. This particular limitation, however, can be overcome through the usage of dendrimers as nanocarriers (Rabiee et al., 2020).

A previous study by Serrama et al. (2015) used carbosilane dendrimers with targeted siRNA to prevent gene expression of a specific protein production in primary astrocytes and cytotoxicity. This study sheds light on the potential of carbosilane dendrimers for drug targeting in the brain.

### 8.5 PLL dendrimers as a potential therapeutic strategy for brain tumors

Poly-l-lysine (PLL) dendrimers are an alternative option to PAMAM dendrimers. Their natural antiangiogenic properties and lower vascularization make them less likely to cause side effects. PLL inhibits tumor development by destroying necrosis and stimulating apoptosis. This contributes to the non-toxic effect PLL dendrimers have on healthy cells, bringing their therapeutic potential to the level of marketed anti-angiogenic drugs (Ryan et al., 2013; Singh et al., 2022). A DOX-loaded PLL dendrimer coupled mainly with folate was synthesised by Jain and colleagues. This dendrimer responded to pH and exhibited antiangiogenic and anticancer activities developed by the researchers (Gauro et al., 2021). There is also the possibility that PLL dendrimers could serve as vehicles for the delivery of anticancer drugs (Gao et al., 2016). Scientists have developed PLL dendrimers that can deliver anticancer drugs to specific tumor sites by binding DOX to the acid-labile bond HSBA (4-hydrazinosulfonylbenzoic acid). Due to the acid-labile binding, only 10% of the drug could be released at a pH of 7.4, while the entire amount of drug could be released at a pH of 5. The results also showed that a decrease in metabolic lability led to an increase in cellular absorption *in vivo*, suggesting that mechanistic targeting of PLL dendrimers may be possible (Kaminskas et al., 2011). Niidome and colleagues developed a G6 PLL dendrimer with PEG-linked penta-alanine or pentaphenylalanine, which made it possible to administer DOX in a targeted manner. The DOX was encased in either a penta-alanine or penta-phenylalanine core, both of which are hydrophobic. Penta-phenylalanine was found to have higher encapsulation efficiency than penta-alanine. DOX was released from the hydrophobic cavity over time as a function of pH. The created dendrimer accumulated more in cancer cells and significantly suppressed tumor development without causing weight loss, suggesting that PLL dendrimers can be targeted in the brain (Malik et al., 2018). Table 2 (Gao et al., 2016; Sharma et al., 2017; Malik et al., 2018; Nh et al., 2016) lists several types of PLL dendrimers.

### 8.6 Poly (propylene imine) (PPI) dendrimers as a brain drug delivery

A new synthesis approach is utilized to develop PPI dendrimers which are hyperbranched macromolecules (Kwan and Leung, 2020). The presence of amino terminal functional groups allows them to be conjugated with ligands such as folate and antibodies to deliver anticancer drugs to specific sites. Dendrimers are deemed as dangerous due to their cationic nature. Wang et al. (2012) utilized a method to circumvent this problem by preparing acetylated PPI dendrimers with 14.2% acylation and 95.3% Doxorubicin/methotrexate (DOX/MTX) loading. Dendrimers with more than 80% acylation were found to be protective against A549 and MCF -7 cells when injected and released over an extended period of time. Another study found that preparation of a folate-free diaminobutane G4 PPI dendrimer ligand would result in lower cytotoxicity. The results underscored that an ETP-loaded dendrimer had long-lasting activity and enhanced site-specific drug delivery (Sideratou et al., 2001). Gajbhiye and Jain conducted research to determine the effectiveness of a polysorbate 80 (P80)-anchored PPI dendrimer (P80-PPI) in transporting DTX (Docetaxel) to the cells in the brain. The study found that DTX-P80-PPI was more cytotoxic than the combination DTX-PPI and the free drug in a human glioma cell line U87MG. Within one week, DTX-P80-PPI demonstrated a possibility of declination in the size of brain tumors (by 50%) in which the significant permeation of P80 through the BBB was linked to this particular finding (Gajbhiye and Jain, 2011). Patel et al. developed a thiamine PPI dendrimer combination that was loaded with Paclitaxel (PTX) so that it could be transported to the brain. In this way, paclitaxel (PTX) could be transported into the brain (PTX-TM-PPI). According to the results of the study, the molecule could prolong the duration of action of drugs while reducing their harmful effects on the body. The PTX-loaded dendrimers and free drug were compared with PTX-TM-PPI, which significantly suppressed tumor formation in a human IMR-32 neuroblastoma cell line. In a separate research study, Patel et al. (2012) examined how effectively PPI dendrimers combined with other ligands, such as concanavalin A (Con A), sialic acid and glucosamine, transported PTX in the brain. According to the cytotoxicity data, the IC50 value of the ligand-anchored PPI dendrimer was three to six times lower than that of the free drug. As the biodistribution was studied, it was found that the ligand-anchored PPI dendrimers showed a higher concentration of the medication in the brain when compared to the free PTX. Not only that, the sialic acid also had better target efficacy than Con A and glucosamine. Table 2 has listed a few examples of PPI dendrimers.

## 9 Dendrimers in brain tumor imaging

Limited accessibility to the BBB is a hurdle that prevents successful identification and treatment of brain tumors. Metastases often occur in advanced stages of cancer and require difficult surgical intervention; therefore, early detection is critical for optimal treatment. The efficacy of nanodiagnostics depends on a well-established imaging technique that can accurately assess the pharmacokinetic profile, bioavailability, tumor neovascularization, uptake by tumor cells and drug and imaging agent release kinetics (Saluja et al., 2021).

A wide range of non-invasive imaging modalities known as molecular imaging have been utilized to visualize, interpret and assess the physiological changes at the molecular/cellular/tissue level to gain insight into the mechanisms of oncogenesis (Zukotynski et al., 2020). Therefore, molecular imaging techniques would be useful for forecasting therapy response, prudently segmenting patients, measuring biodistribution, and determining the drug release profile (Bernsen et al., 2013; Li et al., 2021).

Magnetic resonance imaging (MRI) technique was the first one utilised to detect and diagnose brain tumour lesions (Khan et al., 2020; Almalki et al., 2022; Wu et al., 2022; Yazdan et al., 2022). Meanwhile, gadolinium (Gd)-chelated diagnostic agents are extensively utilized as contrast agents for tumor imaging (Rodríguez-Galván et al., 2020). Currently, there are seven Gd contrast agents approved for clinical use by US-FDA (Heshmatzadeh Behzadi & McDonald, 2022). In the twenty years, ferromagnetic Gd contrast agents coupled with dendrimers have been utilised for the purposes of image intensification, better elimination qualities, and possible targeting while MRI monitoring is being performed (Wu et al., 2022). Dendrimers have been described as nanomedicines in the past. These nanomedicines have the ability to tolerate bigger Gd charges and improve the signal contrast of an MRI contrast agent with nearby tissue *in vivo* (Longmire et al., 2014; McMahon and Bulte, 2018).

In 2011, Han and his co-workers developed a contrast agent for MRI that could be used in the diagnosis of glioma. They came up with the idea of developing PEG-linked PAMAM dendrimers that were loaded with the tumor-selective peptide HAIYPRH (T7). These dendrimers were then connected with diethylenetriaminepentaacetic acid (DTPA) and Gd chelates, which resulted in GdDTPA-PAMAM-PEG-T7. To evaluate the use of dendrimers in the diagnosis of brain tumours, an early brain glioma model was chosen. According to the results, these dendrimers were not able to detect early gliomas. This is because it is difficult to accurately determine the location of a brain tumour as well as its growth due to the challenges posed by the BBB and various pathophysiological conditions (Han et al., 2011).

Recently, Rasouli et al. (2021) investigated the potential of 99 mTc-labelled dendrimer-phenylalanine conjugates in C6 glioma cell lines for brain tumour diagnosis using single photon emission computed tomography (SPECT). The results showed that these dendrimers did not exhibit toxicity in the brain, whereas phenylalanine increased the accumulation and deposition of 99 mTc-labelled dendrimer in brain tumours.

According to the results of a study conducted by Zhao, the resulting G5-NH2 terminal PAMAM dendrimer nanoplatform was further conjugated with PEG, chlorotoxin (CTX) and 3-(4'-hydroxyphenyl)propionic acid-Osu (HPAO). After this step, the remaining terminal amine groups were acetylated and finally labelled with radioactive 131I. *In vivo* studies using the mouse glioma model showed an increase in signal intensity and an anti-cancer effect in SPECT imaging by radionuclides (Zhao et al., 2015).

Subbarayan et al. (2001) prepared two types of soluble dendrimer-based porphyrins (P1 and P2) with a central core of 5,10,15,20-tetrakis [4-(carboxymethyleneoxy)-phenyl]porphyrin (T4CPP) radiolabeled with ^99^mTc for glioma imaging and diagnosis. The results showed that these dendrimers had an efficient imaging potential with a satisfactory diagnostic level in the C6 glioma tumor model. Table 3 consists of a list of dendrimers that can be employed in cancer as contrast agents for MRI, PET and SPECT scans (Han et al., 2011; Huang et al., 2011; Yang et al., 2015; Zhao et al., 2015; Sun et al., 2017; Xu et al., 2019; Rasouli et al., 2021).

**TABLE 3:** Dendrimers used as contrast agents for cancer.

Dendrimer type	Contrast agent	Conjugate	Targeting ligand	Imaging method	Animal mode/Cell line	Ref
Phenylalanine dendrimers	^99^mTc	99 mTc-labeled dendrimer-phenylalanine conjugate	-	SPECT	C6 Glioma cell lines	Han et al. (2011)
G5 PAMAM	^131^I radionuclide	G5-NH_2_ terminated PAMAM-PEG-CTX-HPAO-I131	MMP-2 overexpressing tumor cells	SPECT	Mice glioma cell model	Rasouli et al. (2021)
G3 Dendrigraft poly-L lysine’s	DTPA-Gd	Gd-DTPA-D3-PEG-CTX	Chlorotoxin for MMP-2 receptors	MRI	Mice	Zhao et al. (2015)
Soluble dendrimers	99 mTc	soluble dendrimer- porphyrins (T4CPP)- 99 mTc	--	MRI/PET/SPECT	C6 Glioma cells	Subbarayan et al. (2001)
G2 PAMAM	Macrocyclic Mn (II)	RGD-Au–Mn DENP	Arginine-glycine aspartic acid (RGD) peptide in tumor cells producing αvβ3Integrin	CT and MRI	Mice	Xu et al. (2019)
G3 Poly-l-lysine dendrigraft	DTPA-Gd	SP-PEG-DGL-DTPA-DACHPt	An endogenous neuropeptide known as substance P that binds to the neurokinin-1 (NK-1) receptor	MRI	Mice	Sun et al. (2017)
G5 PAMAM	SPIONs	5NHAc-RGDFe3O4 NPs	RGD peptide for tumour cells that express αvβ3Integrin	MRI	Mice	Yang et al. (2015)
G5 PAMAM	DTPA-Gd	GdDTPA-PAMAM-PEG-T7	Peptide T7 designed to use with cancer cells that express transferrin (Tfr) receptors	MRI	Mice	Huang et al. (2011)
G5PAMAM	131I	131I-labeled dendrimers modified with LyP-1 peptide	dendrimeric nanodevice, radionuclide therapy, antimetastasis therapy of cancer	SPECT	Mice	Song et al. (2020)
Polyglycerol dendrimers	boron-dipyrromethene	boron-dipyrromethene conjugated polyglycerol dendrimers	Fluorescent probe	single-molecule optical imaging	-	Yang et al. (2013)
PAMAM	Sodium dye	Sodium dye conjugated PAMAM dendrimers	sodium-sensitive nanoprobe imaging	Ion imaging	Neural cells	Lamy et al. (2012)

DTPA-Gd, Gadopentetic acid; Gd-DTPA-D3-PEG-CTX, gadopentetic acid-chlorotoxin (CTX) to poly(ethylene glycol) (PEG) coated dendrimers; RGD-Au-Mn DENP, (Arg-Gly-Asp-D-Phe-Lys) peptide modified PEGylated dendrimer-entrapped gold nanoparticles; SP-PEG-DGL-DTPADACHPt, Substance P-PEG- dendrigraft poly-L-lysines Cpmlexed with diethylenetriaminpentaacetic acid and (1,2-diaminocyclohexane platinum(II); 5NHAc-RGDFe3O4 NPs-iron oxide nanoparticles conjugated with Arg-Gly-Asp modified dendrimers.

## 10 Conclusion

According to the results of various animal studies, dendrimers are an interesting candidate for systemic drug delivery for the treatment of brain tumours. Dendrimers and nanotechnology have proven to be the best solution for the treatment of brain tumours. This is because they are an excellent alternative to the conventional chemotherapy available today. Due to their spherical architecture and multivalent periphery, dendrimers can also be used for simultaneous treatment and diagnosis. By using dendrimers, it is possible to reliably target the brain tumour with sustained drug release. In recent years, researchers have become more interested in treating and monitoring brain tumours by taking advantage of the multifunctional properties of BBB, which could improve patient survival. Nanomedicines based on dendrimers, which are highly targeted, effective, biocompatible, and cost-effective, are becoming more popular in the clinic with advances in oncology research. However, dendrimers need further accurate evidence on efficacy, targeting, safety, and mortality concerns to validate them as suitable and viable nanoarchitectures for brain tumour imaging and treatment.

The global outlook for dendrimers in the coming years appears favourable, raising hopes for more successful clinical translation in brain tumours and other malignancies. The ability to provide treatment at the appropriate time depends on rapid diagnosis. This goal may one day be achieved through the use of personalised treatment planning for brain tumours, which aims to improve tumour diagnosis, and drugs that have predictable side effects. Medical advances are shown to have increased patient satisfaction with their life expectancy. Dendrimers are expected to be produced and moved into clinical trials in the next few years.
